# CLSM-guided imaging provides new insights into position-dependent limitations of endodontic disinfection

**DOI:** 10.1007/s00784-026-07091-4

**Published:** 2026-08-26

**Authors:** Sarah Böcher, Rebecca Mattern, Georg Conrads, Andreas Braun, Johannes-Simon Wenzler

**Affiliations:** 1 Department of Operative Dentistry, Periodontology, and Preventive Dentistry, Rheinisch-Westfälische Technische Hochschule (RWTH) University Hospital, Pauwelsstrasse 30, Aachen, 52074 Germany; 2 Division of Oral Microbiology and Immunology, Department of Operative Dentistry, Periodontology, and Preventive Dentistry, Rheinisch-Westfälische Technische Hochschule (RWTH) University Hospital, Pauwelsstrasse 30, Aachen, 52074 Germany

**Keywords:** Disinfection, Endodontics, *Enterococcus faecalis*, Microscopy, Confocal, Root Canal Therapy

## Abstract

**Objectives:**

Insufficient bacterial reduction remains a major cause of endodontic treatment failure. Adequate disinfection is challenging, due to microbial migration into the tubules of the surrounding root dentin. This study aimed to further develop a novel confocal laser scanning microscopy (CLSM) model using LIVE/DEAD staining to provide new insights into endodontic disinfection.

**Materials and methods:**

Thirty human teeth were inoculated with *Enterococcus faecalis* using a previously established protocol (A-Pr2). The area of bacterial colonization and bacterial eradication and the presence of residual bacteria within the irrigated area were evaluated using CLSM-guided imaging with LIVE/DEAD staining, and the sclerosis grade (SCG) of each sample was assessed. The effects of disinfection measures (conventional irrigation plus/minus sonic-activation), position (coronal, medial, apical), and sclerosis grade were analyzed.

**Results:**

Root canal position showed a statistically significant (*p* < 0.05) main effect on bacterial colonization and bacterial eradication, while neither treatment nor sclerosis grade showed statistically significant (*p* > 0.05) main effects. Although the area of bacterial eradication descriptively decreased with increasing sclerosis grade, this trend did not reach statistical significance. Residual bacteria within the irrigated area were more likely in medial and apical than in coronal regions (OR 2.67 and 2.74; both unadjusted *p *< 0.05), with adjusted probabilities of 45.5% (coronal), 69.0% (medial), and 69.6% (apical).

**Conclusions:**

Position within the root canal emerged as the primary determinant of bacterial colonization, extent of bacterial eradication, and risk for persistence of residual bacteria within the irrigated area, highlighting the predominant influence of anatomical location over the investigated treatment variables.

**Clinical relevance:**

Future endodontic disinfection strategies should address position-dependent limitations and aim to reduce the overall bacterial load by considering both the spatial extent (disinfection quantity) and intensity (disinfection quality) of bacterial eradication, rather than penetration depth alone.

## Introduction

Endodontic treatment in primary and recurrent cases is usually carried out because of microbial infections [1]. The success of endodontic treatment significantly correlates with successful bacterial reduction in the infected root canal system. In addition to the mechanical removal of infected hard and soft tissue and the shaping of the root canal for subsequent obturation with a plastic filling material, disinfection (and thus the elimination of bacteria in the root canal system) is therefore a fundamental part of systematic root canal treatment [2, 3]. Between 10^2^ and 10^7^ bacterial cells can be found in an infected root canal system [4], and the migration of these into hard-to-reach areas of the canal — such as the apical delta, accessory lateral canals, and anastomoses, as well as deep into the dentinal tubules of the surrounding root dentin [5] — makes it almost impossible to eliminate them completely [4, 6]. Insufficient bacterial reduction is, in turn, one of the main reasons for the failure of endodontic treatment [4, 7]. Thorough disinfection of all parts of the infected root canal system and of the remaining surrounding dentin is therefore decisive for long-term success.

Irrigation solutions are generally used for this purpose, with sodium hypochlorite (NaOCl) being the most commonly used disinfectant in endodontics due to its antibacterial and tissue-dissolving properties [6, 8]. However, even in combination with mechanical instrumentation of the root canal, on average only 80% of bacteria can be eliminated using thorough and carefully performed chemomechanical treatment [3, 9]. In particular, difficult-to-treat gram-positive bacteria such as *Enterococcus faecalis* have been shown to invade deep into the dentinal tubules and attach to type I collagen fibers [10, 11]. As a consequence, bacteria not reached by the irrigation solutions can lead to recurrent infection, and *E. faecalis* in particular has been shown to be associated with many cases of endodontic failure [7, 12–16].

Sonic-activated and ultrasound-activated irrigation methods have been introduced in recent decades to improve the penetration of conventional irrigation solutions into the surrounding root dentin and eliminate bacterial invaders more effectively [17]. Removal of the smear layer, which can hinder penetration into the dentinal tubules, is considered a prerequisite, and the degree of sclerosis of the teeth (sclerosis grade, SCG) also plays a decisive role both in bacterial colonization and in effective disinfection measures [18].

Recent studies using confocal laser scanning microscopy (CLSM) have already provided impressive visualization of bacteria in dentinal tubules, with the advantages of LIVE/DEAD and other types of functional staining [9, 18–20]. Sample preparation for disinfection studies naturally involves prior bacterial inoculation of the samples. To maximize the depth of bacterial invasion, the samples used in most studies have been pretreated with NaOCl and ethylenediamine tetraacetic acid (EDTA) before inoculation, in order to ensure tubule access [17, 21, 22]. However, the pretreatment process has been reported to fundamentally alter the dentin structure [18] and impedes the evaluation and comparison of the procedures being investigated.

Although CLSM has considerably improved the visualization of bacteria within dentinal tubules, previous studies have mainly focused on the penetration depth of endodontic disinfection measures. Consequently, information on the spatial extent and intensity of bacterial eradication remains limited. To address this knowledge gap, the aim of the present study was to further refine the novel CLSM-guided imaging technique developed by our research group [18, 23] and to go beyond simply assessing the penetration depth of endodontic disinfection measures by introducing an area-based evaluation of bacterial colonization and eradication and a complementary assessment of the presence of residual bacteria. The null hypothesis was that neither disinfection measures (conventional irrigation plus/minus sonic-activation), root canal position (coronal, medial, apical), nor sclerosis grade (SCG) would influence bacterial colonization, the extent of bacterial eradication, and the risk for persistence of residual bacteria within the irrigated area.

## Materials and methods

Tooth preparation, pretreatment (Pr2, without NaOCl), and the inoculation protocol (process A, by overflow), as well as assessment of sclerosis grade (SCG) and CLSM-guided imaging with LIVE/DEAD staining (SYTO 9 and propidium iodide), were performed in accordance with the methodology described in detail in our previous publication [23]. Briefly, thirty freshly extracted, caries-free human teeth with straight root canals were selected. Following crown removal to obtain standardized 12-mm root segments, root canals were prepared to size 50/.05 (ProTaper Gold; Dentsply Sirona, Bensheim, Germany) using NaCl for irrigation between files. Subsequent pretreatment (Pr2) comprised smear layer removal using EDTA (17%) with sonic activation (EDDY; VDW GmbH, Munich, Germany) and irrigation with ultrapure water. No NaOCl was used to avoid alterations in dentin structure associated with increased background fluorescence [18, 23]. Subsequently, specimens were inoculated with *E. faecalis* using the previously established overflow model (process A) and incubated for four weeks [23]. The identical groups published in [23] and repeated below were analyzed to refine the evaluation method and examine the effect of different endodontic disinfection measures:

1. Negative control group (*n* = 5): no bacterial inoculation and no treatment.
2. Positive control group (*n* = 10): bacterial inoculation and no treatment.
3. Conventional irrigation group (conv.) (*n* = 10): bacterial inoculation and conventional irrigation with NaOCl (3%).
4. Sonic-activated irrigation group (EDDY) (*n* = 10): bacterial inoculation and sonic-activated irrigation (5000–6000 Hz; VDW GmbH, Munich, Germany) with NaOCl (3%).

Given the exploratory nature of this study, which was designed as a methodological investigation to further refine the CLSM-guided imaging and analysis approach, no formal a priori sample size calculation was performed.

### Evaluation and assessment of treatment

The CLSM images obtained were analyzed using Dr. Regener Vivo^®^ software (version 7.2; GPSur, Valdivia, Chile). LIVE/DEAD staining allowed visual identification and differentiation of vital and dead bacterial cells in the dentin, as the dye SYTO 9 penetrates all bacteria (intercalating into every DNA/dsRNA fragment), while propidium iodide only reaches the DNA/RNA of membrane-damaged or dead cells [24–26]. Vital bacteria thus showed green fluorescence, while dead bacteria and/or released DNA/RNA additionally showed red fluorescence. The parameters for evaluation were bacterial colonization of the samples (the result of the inoculation), as well as bacterial eradication (the result of the disinfection measures investigated).

The bacterial colonization of the samples with vital bacteria (green fluorescence) was evaluated to determine the presence of *E. faecalis* and to quantify its invasion into the dentinal tubules (positive control group). Bacterial eradication (in the test groups) was evaluated to assess and quantify the efficacy of the disinfection measures evaluated. Only the presence of dead bacteria or released DNA/RNA (red fluorescence) can be regarded as a positive result of the disinfection measures and was therefore used for this evaluation. Additional red fluorescence was caused by altered dentin structures (caused by NaOCl) and had to be carefully distinguished from red fluorescence from dead bacteria and/or released DNA/RNA.

**Fig. 1:**
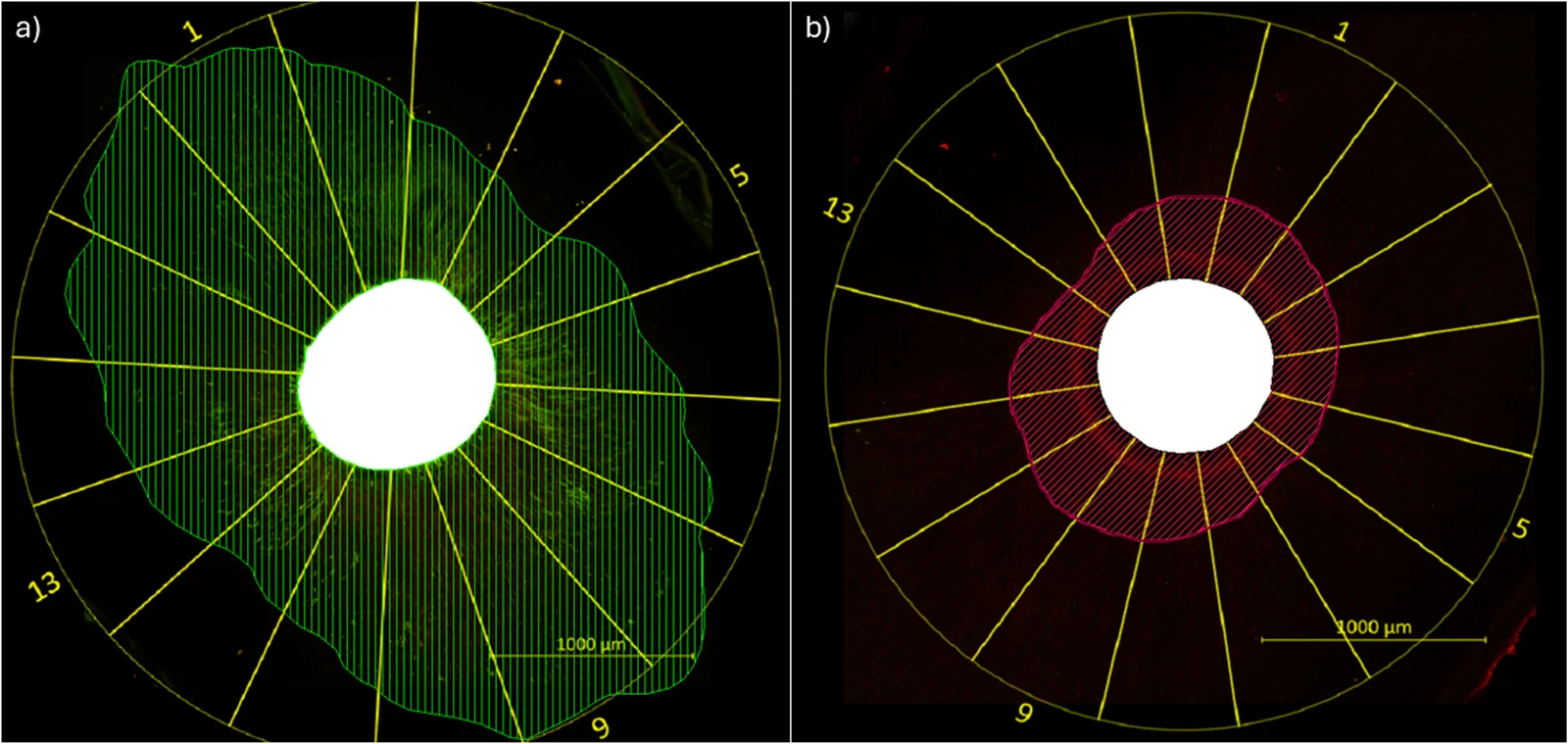
Example illustrating the evaluation method. The area of bacterial colonization (green; positive control group, coronal position, SCG 1) (**a**) and bacterial eradication (red; conventional irrigation group, medial position, SCG 2) (**b**) were quantified for each sample, with the area of the root canal lumen (white) being subtracted

In our previous study [23], both bacterial colonization and bacterial eradication were evaluated on the basis of the individual penetration depths, with the root canal wall serving as reference. This resulted in 16 individual measurement values for bacterial colonization or bacterial eradication per sample, with the mean of the 16 individual measurements per sample being used for further comparison of penetration depths. Due to the butterfly pattern (with the mesiodistal root aspects showing more prominent tubular sclerosis than their buccolingual counterparts [27]), SCG 2 and 3 showed greater penetration depths (for bacterial colonization and bacterial eradication) in the buccolingual than in the mesiodistal direction (butterfly penetration pattern), making meaningful comparison (and interpretation) of the data more complicated. Further slight inaccuracies resulted from the fact that only 16 individual values were recorded for each sample, and for that reason a second evaluation method was applied. For this purpose, the area of bacterial colonization and eradication for each sample was additionally evaluated and used for subsequent analysis (Fig. 1).

In addition, the presence of residual vital bacteria (green fluorescence) within the irrigated area was evaluated by subdividing each tooth slice into 16 equally sized segments (S1–16). For each segment, the presence of residual vital bacteria was assessed using a binary yes-or-no decision, and the number of segments exhibiting residual bacteria was then related to the total number of evaluable segments per tooth slice.

### Statistical analysis

Statistical analysis was performed using R (version 4.5.2; R Foundation for Statistical Computing, Vienna, Austria). Linear mixed-effects models were fitted, with the area of bacterial colonization or bacterial eradication as the dependent variable. Treatments (conventional irrigation [conv.], sonic-activated irrigation [EDDY]), position (coronal, medial, apical), and degree of sclerosis (SCG) were included as fixed effects, with all possible two-way interactions. A random intercept for tooth was included to account for the non-independence of measurements. As the raw data for bacterial eradication showed a pronounced right-skewed distribution, the values were log-transformed prior to analysis. Fixed effects (main effects and two-way interactions) were statistically assessed using type III analysis of variance with Kenward–Roger approximation for degrees of freedom. Estimated marginal means (EMMs) with 95% confidence intervals were derived from the fitted models. For bacterial eradication, EMMs are reported as geometric means (GMs). Post-hoc pairwise comparisons were performed if appropriate using model-based *t*-tests on EMMs, with *p* values adjusted for multiple testing using Tukey’s method. Statistical significance was set at *p* < 0.05.

The presence of residual vital bacteria within the irrigated area was analyzed using beta-binomial mixed-effects models with a logit link function to account for overdispersion in binomial data. Treatment, position, and degree of sclerosis (SCG) were included as fixed effects, and a random intercept for tooth was specified, analogous to the models described above. Fixed effects (main effects and two-way interactions) were statistically assessed using likelihood ratio tests by comparing nested models. Model-based estimated marginal means (EMMs) on the response scale were calculated to obtain adjusted probabilities of residual bacterial presence. Post-hoc pairwise comparisons were performed if appropriate using model-based *z*-tests on EMMs, with *p* values adjusted for multiple testing using Tukey’s method. Statistical significance was set at *p* < 0.05.

## Results

### Evaluation of bacterial colonization

A statistically significant interaction between position (coronal, medial, apical region) and SCG was not observed (F(4, 16.5) = 0.51, *p* = 0.73). A statistically significant main effect of position was observed (F(2, 16.3) = 7.02, *p* = 0.006), but the main effect of SCG was not statistically significant (F(2, 14.7) = 2.65, *p* = 0.10).

Accordingly, SCG-adjusted estimated marginal means showed a position-dependent gradient for the area of bacterial colonization from coronal to apical (coronal > medial > apical). Post-hoc comparisons indicated a statistically significantly larger area of bacterial colonization at the coronal position in comparison with the medial and apical positions (both *p* < 0.05), while no significant difference was observed between the medial and apical positions (*p* > 0.05; Table 1). Although the main effect of SCG was not statistically significant (*p* > 0.05), bacterial colonization descriptively decreased from SCG 1 to SCG 3 (SCG 1 > SCG 2 > SCG 3).

**Table 1:** SCG-adjusted estimated marginal means (EMMs) for the area of bacterial colonization (mm^2^) across positions coronal to apical

Position	EMMs	Lower 95% CI	Upper 95% CI
Coronal ^a^	4.04	2.84	5.23
Medial ^b^	1.77	0.40	3.14
Apical ^b^	1.14	0	2.37

Values are presented as EMMs derived from the linear mixed-effects model with 95% confidence intervals (95% CI). Lower confidence limits extending below zero were truncated at zero, as negative values are not biologically meaningful. Different superscript letters indicate statistically significant differences between positions (*p* < 0.05, Tukey-adjusted)

*SCG* Degree of sclerosis, *EMM* Estimated marginal means

**Fig. 2:**
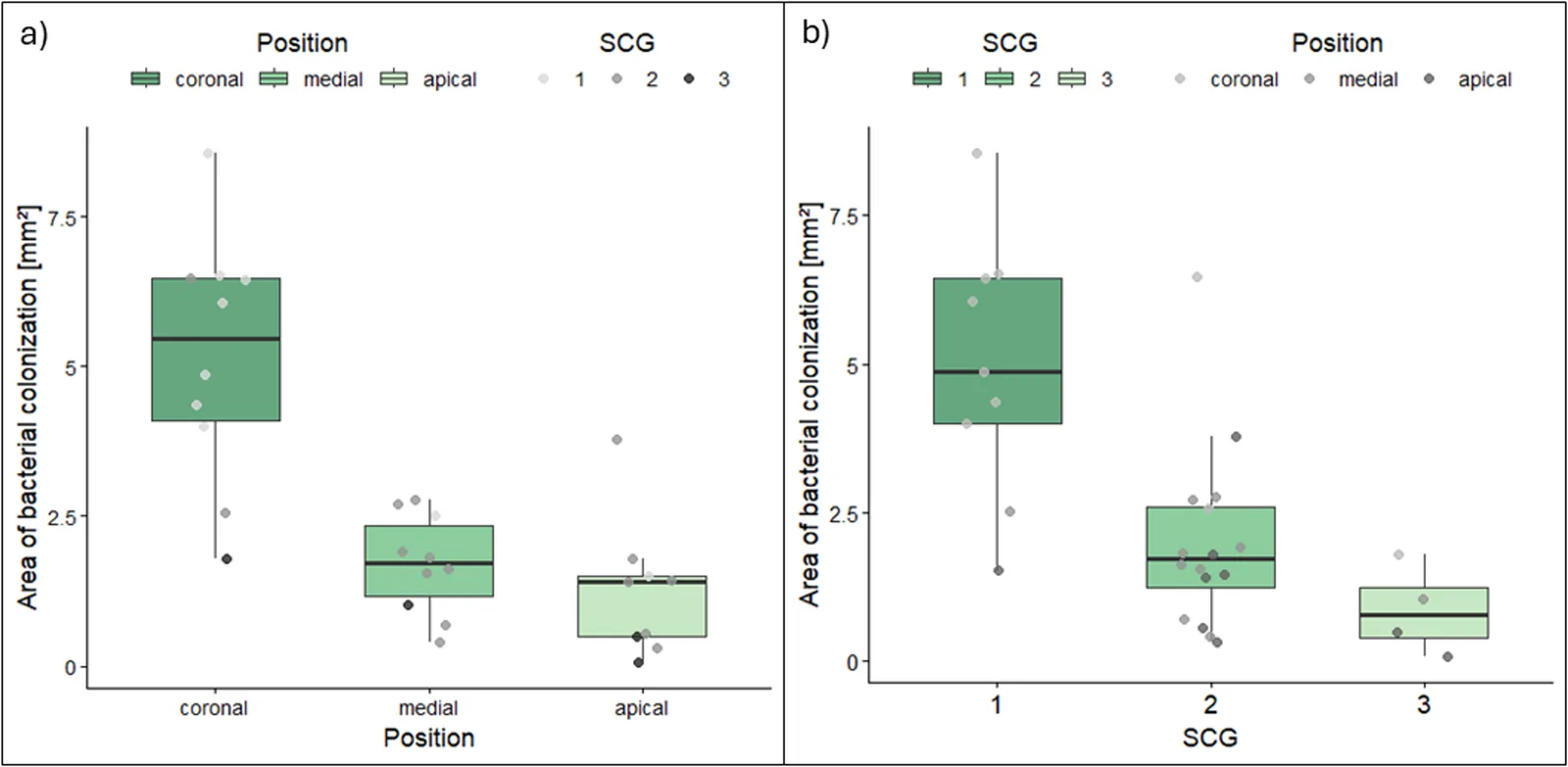
Box plots for the areas of bacterial colonization measured (mm²). The box plots illustrate the distribution of the area of bacterial colonization measured across positions (**a**) and the degree of sclerosis (SCG) (**b**). Individual data points represent measured values

The distribution of the area of bacterial colonization across positions and degrees of sclerosis (SCG) is illustrated in Fig. 2; descriptive statistics for the raw data are provided in Table 2.

**Table 2:** Descriptive statistics for the area of bacterial colonization measured in mm^2^

	Position			SCG			All
	Coronal	Medial	Apical	1	2	3	
Median	5.46	1.72	1.41	4.87	1.72	0.76	1.81
Minimum	1.78	0.39	0.06	1.50	0.31	0.06	0.06
Maximum	8.56	2.77	3.77	8.56	6.46	1.78	8.56
IQR	2.37	1.19	1.02	2.45	1.36	0.84	2.60

Values represent raw, untransformed data

*IQR* Interquartile range, *SCG* Degree of sclerosis

### Evaluation of the efficacy of disinfection measures (bacterial eradication)

No statistically significant two-way interactions between the fixed effects were observed (all *p* > 0.05). A statistically significant main effect of position was detected (F(2, 34.36) = 3.55, *p* = 0.040), whereas neither the main effect of SCG (F(2, 41.36) = 2.69, *p* = 0.080) nor treatment (F(1, 15.91) = 1.22, *p* = 0.285) reached statistical significance.

Accordingly, SCG-adjusted and treatment-adjusted estimated marginal means (EMMs; reported as geometric means [GMs]) showed a position-dependent gradient for the area of bacterial eradication (coronal > medial > apical). Post-hoc pairwise comparisons revealed a statistically significantly larger area of bacterial eradication at the coronal position in comparison with the apical position (approximately threefold higher, ratio = 2.98, *p* = 0.033), whereas no statistically significant differences were observed between the coronal and medial or between the medial and apical positions (both *p* > 0.05; Table 3). Although the main effect of SCG was not statistically significant (*p* > 0.05), the area of bacterial eradication descriptively decreased with increasing degree of sclerosis (SCG 1 > SCG 2 > SCG 3).

**Table 3:** SCG-adjusted and treatment-adjusted estimated marginal means (EMMs), reported as geometric means (GMs), for the area of bacterial eradication (mm^2^) across positions coronal to apical

Position	GMs	Lower 95% CI	Upper 95% CI
Coronal ^a^	3.23	1.54	6.78
Medial ^a, b^	1.25	0.81	1.94
Apical ^b^	1.08	0.69	1.70

Values are presented as estimated marginal means (EMMs) derived from the linear mixed-effects model fitted on log-transformed data. They are therefore reported as geometric means (GMs); 95% confidence intervals (95% CI) are shown. Different superscript letters indicate statistically significant differences between positions (*p* < 0.05, Tukey-adjusted)

**Fig. 3:**
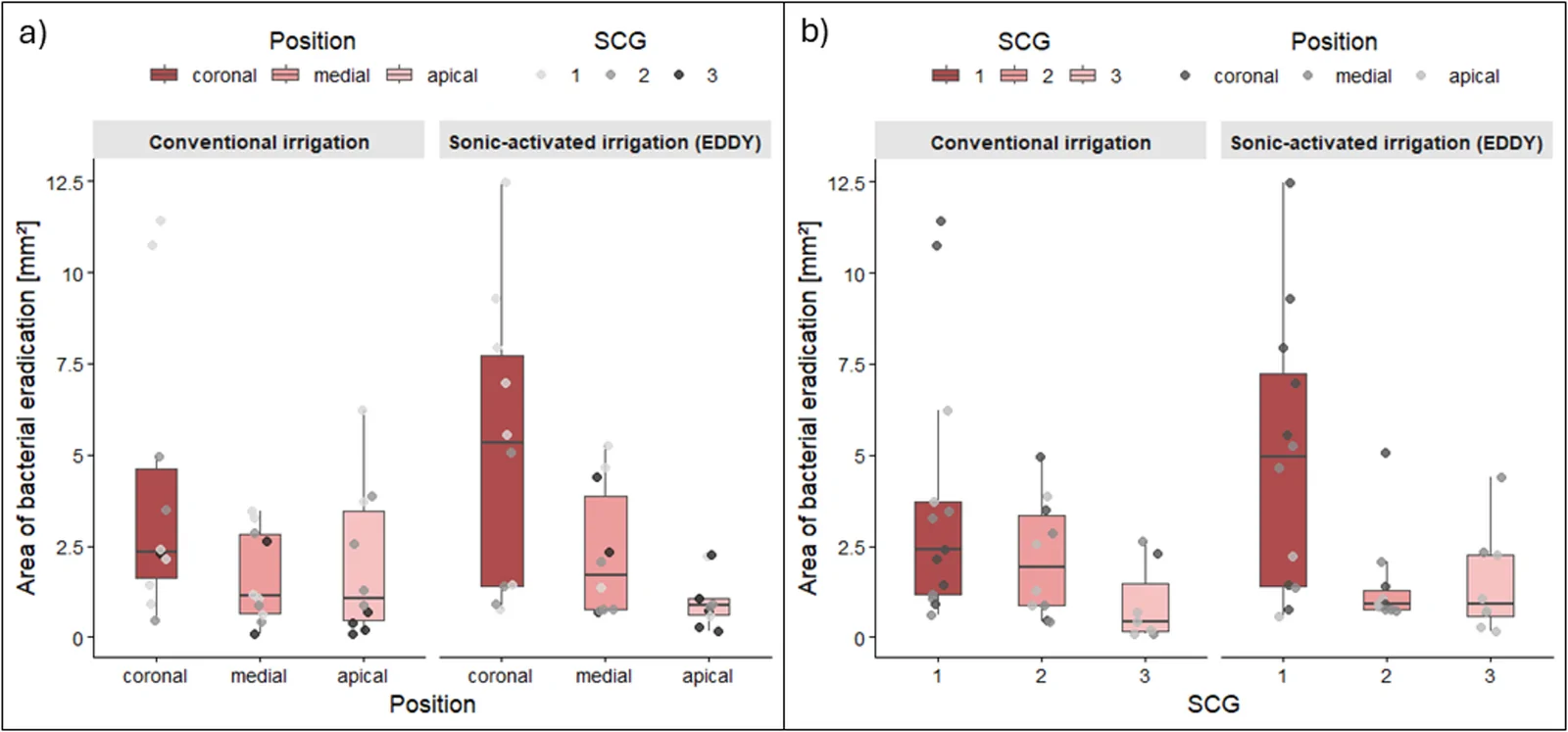
Box plots for the area of bacterial eradication (mm²). The box plots illustrate the distribution of the measured area of bacterial eradication across positions (**a**) and the degree of sclerosis (SCG) (**b**). Individual data points represent measured values

The distribution of the area of bacterial eradication across positions and degrees of sclerosis (SCG) is illustrated in Fig. 3; descriptive statistics for the raw data are provided in Table 4.

**Table 4:** Descriptive statistics for the area of bacterial eradication measured in mm^2^

	Conventional irrigation (conv.)						
	Position			SCG			All
	Coronal	Medial	Apical	1	2	3	
Median	2.35	1.12	1.07	2.40	1.93	0.41	1.79
Minimum	0.46	0.09	0.10	0.60	0.43	0.09	0.09
Maximum	11.43	3.46	6.23	11.43	4.96	2.65	11.43
IQR	2.99	2.15	2.96	2.56	2.49	1.34	2.68

	Sonic-activated irrigation (EDDY)						
	Position			SCG			All
	Coronal	Medial	Apical	1	2	3	
Median	5.32	1.71	0.88	4.96	0.91	0.90	1.37
Minimum	0.77	0.69	0.16	0.59	0.71	0.16	0.16
Maximum	12.48	5.25	2.25	12.48	5.08	4.39	12.48
IQR	6.31	3.13	0.43	5.80	0.53	1.69	3.84

Values represent raw, untransformed data

*IQR* Interquartile range, *SCG* Degree of sclerosis

### Evaluation of residual bacteria

No statistically significant two-way interactions between position, treatment, and SCG were detected (all *p* > 0.05) and such interactions were therefore not retained in the final model. The beta-binomial mixed-effects model revealed a statistically significant main effect of position (χ²(2) = 6.31, *p *= 0.043) on the presence of residual bacteria within the irrigated area. In comparison with the coronal position, both the medial (OR = 2.67, unadjusted *p *= 0.020) and apical positions (OR = 2.74, unadjusted *p *= 0.034) showed higher odds of residual bacterial presence. No statistically significant main effects were observed for treatment (conventional irrigation [conv.] vs. sonic-activated irrigation [EDDY]; χ²(1) = 0.16, *p* = 0.686) or SCG (χ²(2) = 2.05, *p* = 0.360). Model-based adjusted probabilities demonstrated an increasing probability of residual bacterial presence from the coronal (45.5%) to the medial (69.0%) and apical positions (69.6%). Pairwise post-hoc comparisons between positions did not reach statistical significance (all *p* > 0.05) after adjustment for multiple testing.

## Discussion

**Fig. 4:**
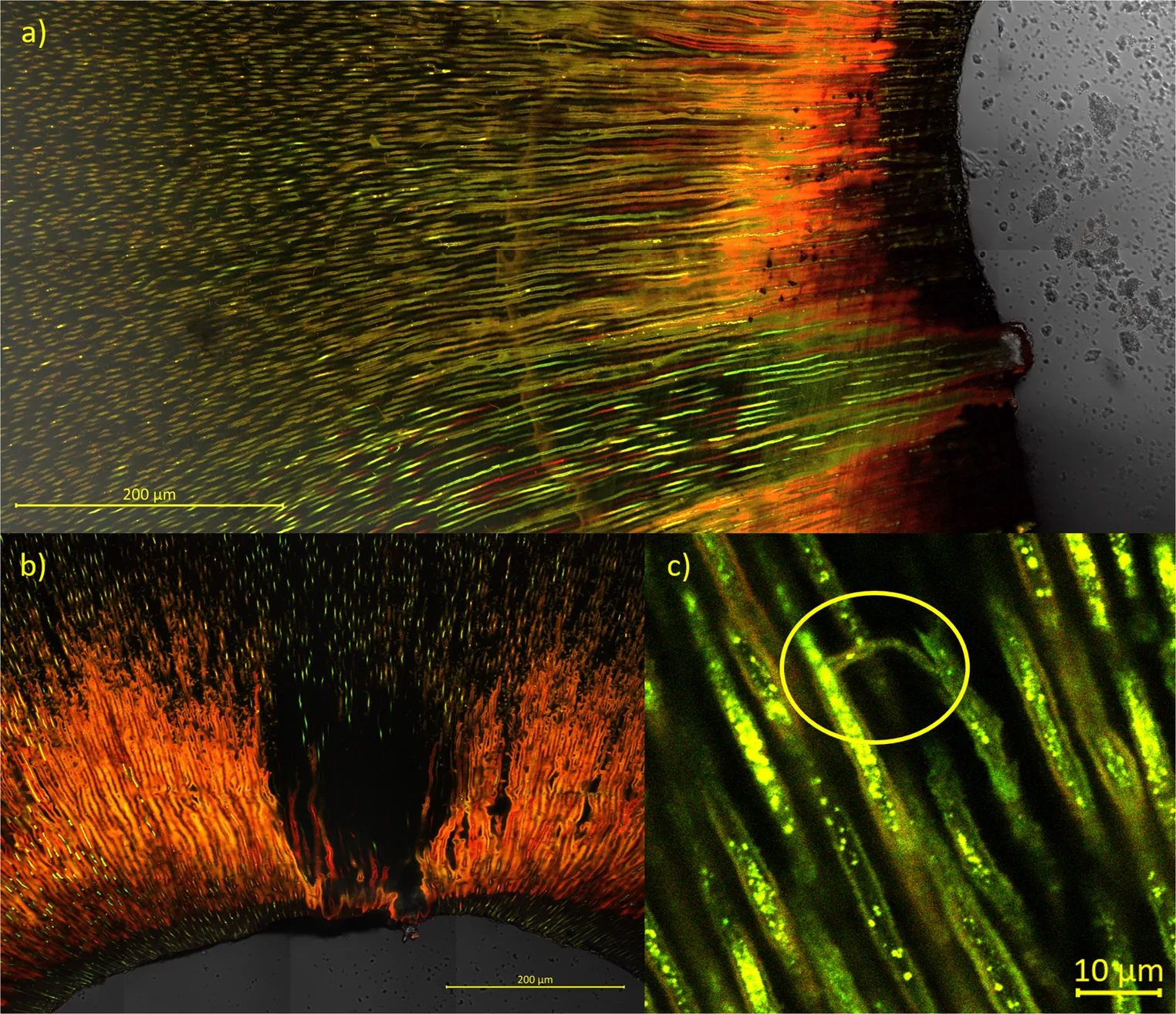
Representative images illustrating reasons for residual bacteria (visualized using confocal laser scanning microscopy (CLSM) and LIVE/DEAD staining). **a**,** b) **Irrespective of the treatment protocol, some dentin areas may not be reached by disinfection measures if the entrance of the dentinal tubules is blocked by local histological irregularities and/or debris — (**a**) with sonic-activated irrigation (EDDY) and **b**) in the conventional irrigation group. **c) **Vital bacteria may also persist in hard-to-reach sections of the dentin, such as anastomoses of dentinal tubules (yellow circle)

Research on endodontic disinfection measures is still necessary, since on average only 80% of the bacteria in the root canal system can be eliminated by thorough and carefully performed chemomechanical treatment [3, 9]. This limited efficacy is mainly due to the complex anatomy of the root canal system and the microstructure of the surrounding dentin. In particular, unevenness of the root canal walls as well as irregular canal anatomies, ramifications, isthmuses, branching of the apical delta, and the location of the foramina pose major difficulties [5, 28, 29]. Mechanical instrumentation of the root canal cannot fully reach all parts of the root canal wall, leaving about 17–25% untouched or incompletely prepared [30]. Untouched or incompletely prepared areas, as well as areas covered by debris consisting of tissue residues, bacteria, and dentin chips, may also impede disinfection of the surrounding dentin [31]. Thus, some dentin areas may not be reached by disinfection measures because the entrance of the dentinal tubules is blocked by debris, resulting in the persistence of vital bacteria in such “closed” dentinal tubules (Fig. 4a, b).

In addition, dentinal tubules form a complex three-dimensional network of radially oriented microchannels with numerous fine lateral branches and anastomoses [32, 33]. This intricate architecture leads to hard-to-reach dentin sections and constrictions that provide protected niches for bacteria [34]. Furthermore, tubule diameter and density vary depending on root canal position and dentin depth, generally decreasing from coronal to apical positions and with increasing distance from the root canal lumen [32, 33, 35]. Conversely, recent research has shown that microbranch density significantly increases in areas with lower tubule density [33]. These anatomical characteristics may explain why root canal position emerged as the primary determinant of bacterial colonization, the extent of bacterial eradication, and the risk for persistence of residual bacteria within the irrigated area. In the present study, high-resolution CLSM imaging showed that bacteria may invade not only the primary tubules, but — depending on their size — also lateral branches and anastomoses (Fig. 4c) that may not be reached by disinfection measures. Therefore, dentinal tubules are considered an important bacterial reservoir that may contribute to persistent or recurrent infection during and after endodontic treatment [34].

Since anatomical intricacies in the root canal system, including dentinal tubules, are closed-ended cavities, bulk irrigant flow (the most effective transport mechanism) is mainly confined to the main root canal and adjacent wider areas [34], whereas irrigant penetration into dentinal tubules depends predominantly on diffusion. As diffusion is an inherently slow process and dentin permeability decreases with smaller tubule diameters, irrigant delivery becomes increasingly limited in deeper dentin layers and more apical regions [34, 36]. Furthermore, irrigation solutions such as NaOCl are continuously consumed through reactions with dentin, biofilm, and organic substrates, leading to a progressive reduction in antimicrobial efficacy during penetration into dentinal tubules [36]. As a result, bacterial eradication may not be achieved in deeper dentin layers if sufficient concentrations cannot be reached and maintained at the target site for an adequate exposure time [34, 37].

To improve the effectiveness of disinfection measures in hard-to-reach canal sections and the surrounding root dentin, various adjuvant treatment methods have been proposed, such as ultrasound-activated or sonic-activated irrigation methods, hydrodynamic irrigation methods, and laser-based therapy options. For correct interpretation, it is important to distinguish between studies that investigate the effectiveness of disinfection measures or debris removal in the root canal itself and in hard-to-reach canal sections, on the one hand, and studies that evaluate the penetration depth into the surrounding root dentin on the other. Studies have shown that debris, biofilm, and residual tissue can be removed much more effectively from the root canal and associated grooves or lateral canals using adjunctive irrigation methods of this type [38, 39]. However, that was not an objective of this study. The present study focused on evaluating the extent of penetration (or, more precisely, the effectiveness) of disinfection measures into the surrounding root dentin; the removal of debris was not an objective of this study and was therefore not evaluated.

Over time, various methods have been evaluated for assessing and quantifying the penetration of endodontic disinfection measures into the surrounding root dentin. These included histological methods [40], microbial cultivation methods [41–44], dye penetration [17, 27, 45–48] and discoloration tests [49–51], and scanning electron microscopy (SEM) imaging [21]. Most of these studies have provided only limited information on only a few selected depths (histological or culture methods) or no information on the actual efficacy of endodontic disinfection measures (dye penetration or discoloration tests). Their advantages and disadvantages have already been discussed in detail previously [23]. More recent studies have used SEM imaging to visualize and quantify bacteria at different depths in the dentin [21], with the disadvantage that SEM imaging does not provide any information about the vitality of the bacteria. CLSM imaging in combination with LIVE/DEAD staining, on the other hand, makes it possible to distinguish between vital and dead bacterial cells and thus to assess the actual effectiveness of disinfection measures [18, 23]. However, only a few studies to date have used CLSM imaging and LIVE/DEAD staining [9, 19, 20, 52].

Al Shahrani et al. (2014), for example, used CLSM imaging for illustrative purposes only and reported that after irrigation with NaOCl, red fluorescence from dead bacteria was only visible on the surface layer, while deeper layers exhibited green fluorescence of vital bacteria; quantitative analysis was not performed [52]. Neelakantan et al. (2015) evaluated the ratio of dead bacterial cells (ratio of red fluorescence to total fluorescence) at depths of 200 μm and 400 μm and reported a 64.79% rate of dead cells at 200 μm and 43.27% at 400 μm after irrigation with NaOCl [19], while Zeng et al. (2018) used a similar approach to evaluate the ratio of dead bacterial cells at depths up to 150 μm from the root canal [20]. Another study by Vatkar et al. (2016) measured the penetration depth achieved by disinfection measures and found a zone of dead bacteria to a depth of 88–110 μm for NaOCl [9].

However, no studies using CLSM imaging and only a few studies using dye penetration [27, 45] or discoloration tests [49] have evaluated the area affected by disinfection measures, which makes comparison difficult. Studies by Paqué et al. (2006) and Macías et al. (2017) used dye penetration tests with Patent Blue V [27] or Chinese ink [45] and evaluated the percentage of the stained area relative to the total area of the root dentin; however, they did not provide absolute values [27, 45]. Only one study, by Giardino et al. (2017), which used a discoloration test with copper sulfate solution, reported areas of 108,592 μm² (coronal), 73,757 μm² (medial) and 39,715 μm² (apical) [49], with these values being significantly lower than those observed in the present study. Due to differences in tooth size, it may be useful to evaluate the area of bacterial eradication relative to the total area of the root dentin, as in Macías et al. (2018) [45], and this should be considered in future studies.

None of these studies on the area affected by disinfection measures compared conventional with sonic-activated irrigation methods. Only a few studies that used penetration depth values have compared the efficacy of conventional and sonic-activated irrigation, including studies that used dye penetration tests with methylene blue [17, 47, 48] or discoloration tests [51] — with divergent results. While some studies have reported no statistically significant differences (*p* > 0.05) between conventional and sonic-activated irrigation (EDDY) [17, 47], others have described statistically significantly (*p* < 0.05) higher penetration depths for sonic-activated irrigation using the Endo Activator (Dentsply Sirona, Bensheim, Germany) in comparison with conventional needle irrigation and manual dynamic activation [48, 51]. The Endo Activator, however, has a lower frequency of 2000–3000 Hz and is therefore not fully comparable.

Only one study using CLSM imaging compared conventional with sonic-activated irrigation (EDDY) and concluded that both were less effective in apical positions. Neither system was able to eliminate bacteria deep within the dentinal tubules, but sonic-activated irrigation (EDDY) showed statistically significantly (*p* < 0.05) more intratubular dead bacteria in all positions [20].

In the present study, no statistically significant (*p* > 0.05) main effect of the disinfection measure (conventional irrigation vs. sonic-activated irrigation [EDDY]) was observed, indicating that the area of bacterial eradication did not differ significantly between conventional and sonic-activated irrigation (EDDY) when averaged across positions and sclerosis grade (SCG). In addition, no statistically significant interactions between treatment and position or treatment and sclerosis grade (SCG) were detected (all *p* > 0.05), suggesting that the effect of the disinfection measure did not vary across positions or sclerosis grade (SCG). In contrast, a statistically significant main effect of position was observed, with model-based estimates indicating a position-dependent gradient of the area of bacterial eradication (coronal > medial > apical). Although the sclerosis grade (SCG) did not show a statistically significant (*p* > 0.05) main effect, estimated marginal means (EMMs) revealed a descriptive trend toward a lower area of bacterial eradication with increasing SCG (SCG 1 > SCG 2 > SCG 3). This trend did not reach statistical significance and should therefore be interpreted with caution. Overall, these findings suggest that position is the primary factor influencing the area of bacterial eradication in the experimental conditions of the present study. Unlike root canal position, which is a fixed anatomical characteristic, the sclerosis grade (SCG) cannot be reliably assessed in clinical practice. Therefore, patient age may represent a useful surrogate parameter because of its well-established association with dentinal sclerosis [53].

The fact that statistically significant (*p* < 0.05) differences between conventional and sonic-activated irrigation (EDDY) have been observed for the analysis of average penetration depths [23], but not for the analysis of the area of bacterial eradication (present study), could be due to wide differences in the position-dependent individual penetration depth values per sample (due to the butterfly pattern). As previously reported [23], both groups showed greater penetration depths in the buccolingual direction than in the mesiodistal direction (butterfly penetration pattern). This effect was more pronounced in the conventional irrigation group, while sonic-activated irrigation (EDDY) resulted in a more homogeneous radial (omnidirectional) irrigation pattern. If the differences within the individual position-dependent penetration depth values are not “evenly” distributed between the different groups — i.e., for conventional irrigation, there may be few areas with large penetration depths but many areas with only very shallow depths, so that the average penetration depth is considerably lower, whereas more uniform values are obtained for sonic-activated irrigation (EDDY), resulting in a greater average penetration depth — then the average penetration depth values may not be suitable for subsequent analysis and may lead to overestimation of the differences between the disinfection measures.

Since bacterial colonization and eradication within the surrounding root dentin appear anisotropically distributed as a result of dentinal tubule morphology (butterfly pattern) [27, 53], this inherent structural anisotropy poses a fundamental challenge for linear penetration depth measurements. Importantly, this limitation is not primarily related to the number of radial measurements obtained, but rather to the anisotropic spatial distribution itself. Linear depth measurements along a given measurement axis are restricted to discrete radial locations and associated with considerable circumferential variability. Therefore, summary statistics such as mean or median penetration depths provide only a partial representation and may not adequately reflect the complex spatial distribution of bacterial colonization or eradication within the root dentin. Additionally, direct comparisons across studies may be complicated when the anatomical location of the maximum penetration depth is not standardized or reported, providing an additional rationale for complementing linear penetration depth measurements with area-based assessment. Area-based assessment approaches may also provide complementary information when bacterial colonization is spatially heterogeneous. Since oral biofilms are increasingly recognized as highly structured microbial communities in which spatial organization (biogeography) represents an important biological feature [54, 55], this provides a biological rationale for complementing conventional penetration depth measurements with area-based assessment. Further studies are warranted to evaluate the reproducibility, biological relevance, and potential clinical significance of these complementary outcome measures.

In this regard, the use of a monospecies biofilm model (*E. faecalis*) may be considered a limitation of the present study, as monospecies biofilms cannot fully reproduce the microbial interactions, extracellular matrix composition, and spatial organization of multispecies biofilms associated with endodontic infections [34, 37, 56, 57]. In addition, biofilm maturity is known to substantially influence the susceptibility to endodontic disinfection measures [34, 37, 56] and therefore a four-week biofilm maturation period was chosen to establish a mature and structurally organized biofilm model. Nevertheless, further studies using mature multispecies biofilms are warranted to validate the introduced outcome measures and further refine the proposed CLSM methodology.

Additionally, there is a considerable difference between the mere depth of penetration of the disinfection measures into the surrounding dentin and the amount of bacteria they ultimately eliminate at different depths. For example, conventional and sonic-activated irrigation (EDDY) may reach the same penetration depth, but conventional irrigation may eliminate relatively few bacteria, while sonic-activated irrigation (EDDY) may eliminate a significantly larger number (with a different disinfection intensity). Given the impossibility of completely eliminating microorganisms from the root canal system, there is consensus that bacterial load reduction in the root canal system to a level below that which is pathologically relevant and is controllable by the immune system is essential for long-term treatment success [20, 58]. Consequently, reducing the total bacterial load is of major importance, with the combination of penetration depth and disinfection intensity playing a more important role than penetration depth alone. For future studies on disinfection measures, it would therefore be important to establish a uniform definition of the “depth of effective disinfection” and to focus on the combination of disinfection depth and intensity. Some of the studies that used CLSM imaging have in fact evaluated the effectiveness of disinfection measures by analyzing red fluorescence relative to total fluorescence [19, 20]. The problem here is that possible background fluorescence was not taken into account in these studies. In the present study, however, significant background fluorescence was observed — particularly red fluorescence — which appeared to be attributable to changes in the dentin structure caused by NaOCl, as previously described [18]. Since no background fluorescence has been observed [52] or reported [20] in other studies using CLSM imaging — although the same dyes (SYTO 9 and propidium iodide) were used for LIVE/DEAD staining [9, 19, 20, 52]) and it may even be enhanced depending on the disinfection method used — this could have led to an overestimation of the effectiveness of the disinfection measures. Although objective analyses of fluorescence intensities could represent a promising evaluation strategy, strategies need to be developed to standardize the varying levels of background fluorescence that may occur depending on the disinfection measures used.

In addition to its highly desirable properties in endodontic disinfection, NaOCl appears to be associated with the undesirable side effect of decomposing the collagen matrix in mineralized dentin, thus altering the dentin structure depending on exposure time and concentration [59]. In a previous publication, our group already demonstrated that red fluorescence (background fluorescence) also occurred in negative control samples that had been pretreated with NaOCl (additionally to EDTA) [18], being even more pronounced with frequent use of NaOCl. For this reason, pretreatment with EDTA alone (Pr2) was used as the preferred method in the present study.

Alterations in dentin structure caused by NaOCl have already been demonstrated using SEM imaging [60, 61]. Due to its small size, the hypochlorite anion can penetrate the water compartments of apatite-encapsulated collagen fibrils and degrade the collagen molecules, resulting in a mineral-rich, collagen-poor “ghost mineral layer” with reduced flexural strength [62, 63]. It has been shown that EDTA even accelerates the destructive action of NaOCl [60, 64] and that the combination of EDTA and NaOCl causes progressive dissolution of dentin at the expense of peritubular and intertubular areas, due to their synergic action [65, 66]. When EDTA is used alone, it removes calcium ions from the mineralized tissue (demineralization of inorganic components), with the organic matter being the limiting factor for further dissolution [60, 63]. Subsequent irrigation with NaOCl removes the organic matrix of the dentin and facilitates the exposure of the inorganic matrix, thus enabling further demineralization by EDTA [60, 67]. The combined use of NaOCl and EDTA therefore leads to excessive erosion of the intertubular and peritubular dentin, with exposure of the superficial collagen fibers in the intertubular dentin. Penetration into the dentinal tubules also causes changes in the peritubular dentin (more pronounced in superficial layers and decreasing with increasing depth), leading to enlargement, funnel-shaped expansion, and partial fusion of the dentinal tubules [60, 62, 68].

**Fig. 5:**
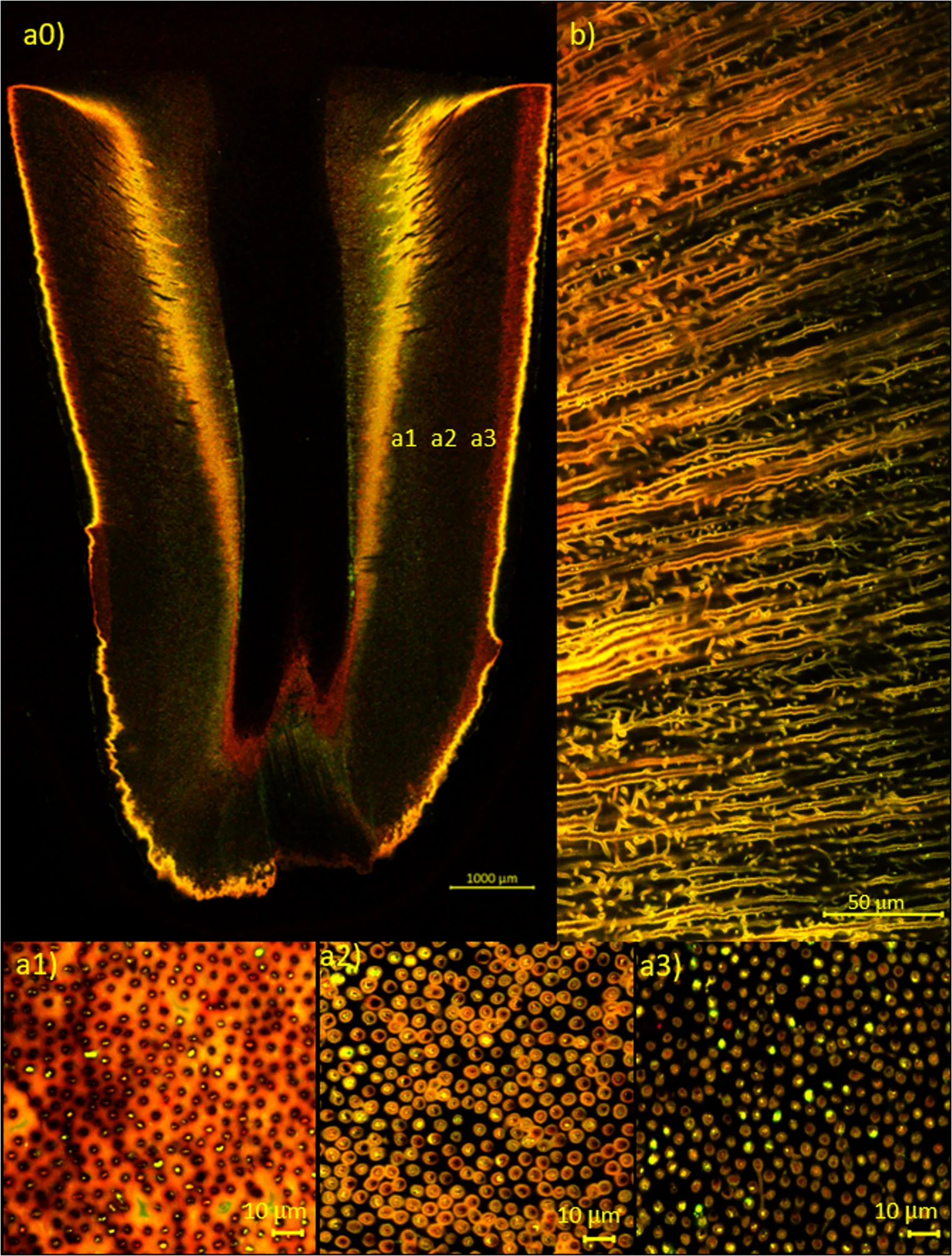
Representative images illustrating changes in the dentin ultrastructure (visualized using confocal laser scanning microscopy (CLSM) and LIVE/DEAD staining), with pretreatment involving NaOCl (in contrast to the present study protocol). **a0**) A longitudinal section of the root canal (scale bar 1000 μm). **a1–3) **Detailed images obtained at different depths in the dentin (with the depth increasing from a1 to a3), with dentinal tubules cut perpendicular to their longitudinal axis. **a1) **Dentin adjacent to the root canal shows clearly visible changes in the dentin ultrastructure (intertubular dentin appears to be altered, with red-orange staining and with enlarged and partly coalesced dentinal tubules), with vital bacteria (green fluorescence) still visible in some tubules. **a2) **In deeper layers of the dentin, the staining is significantly less pronounced and limited to the peritubular dentin, with some tubules still filled with vital bacteria. **a3) **Staining that decreases with increasing depth and a rising number of vital bacteria can be seen inside the tubules. **b) **Dentinal tubules cut longitudinally, with staining of the peritubular dentin (surrounding the branches and microbranches) gradually decreasing with increasing depth

The observed increased background fluorescence may therefore also be explained by the synergic effect of NaOCl and EDTA. Figure 5 shows an example of a longitudinal section of a tooth that had been pretreated with NaOCl (in addition to EDTA, unlike the present study protocol), as well as detailed images obtained at different depths in the dentin. The dentin adjacent to the root canal shows clearly visible changes in the dentin ultrastructure — the intertubular dentin appears to be altered (red-orange staining), with enlarged and partly coalesced dentinal tubules) (Fig. 5, a1). In deeper layers of the dentin, the staining is significantly less pronounced and limited to the peritubular dentin (Fig. 5, a2), decreasing with increasing depth (Fig. 5, a3). The demineralized dentin areas and the resulting increased porosity of the dentinal structure — superficial layer of intertubular dentin and superficial layer within the dentinal tubules (peritubular dentin) surrounding the branches and microbranches — may enable or promote penetration and retention of the LIVE/DEAD dyes in the weakened dentinal matrix, resulting in the clearly visible red-orange fluorescence (decreasing with increasing depth). Since studies have shown that dentin samples show different susceptibility to EDTA possibly due to differences in root dentin calcification [60], it can be assumed that younger, less mineralized dentin (SCG 0–1) is more susceptible to the synergistic use of EDTA and NaOCl. Further studies are needed in order to understand the associated changes in dentin structure and clarify how these affect tooth structure, strength, and longevity [60].

In our previous study [18], the penetration depth proved to be strongly dependent on the degree of sclerosis and thus on dentin density. A “butterfly shape” of the sclerotic dentin is typical for tubular sclerosis of root dentin, with the mesiodistal direction showing more intratubular calcification than the buccolingual counterparts, as described by Vasiliadis et al. (1983) [53]. Bacterial invasion and penetration of disinfection measures are particularly prevalent in areas with open tubules and are therefore more pronounced in the buccolingual than in the mesiodistal direction (butterfly penetration pattern) — a pattern that has been described as a “barbell shape” in earlier studies [27]. No main effect of SCG was observed in the present study. However, both the area of bacterial colonization and bacterial eradication descriptively decreased with increasing degree of sclerosis. Given the exploratory nature of this study, further studies with a larger sample size will be needed to confirm the present findings and to further investigate the effect of SCG in different positions. In the present study, position emerged as the primary factor not only for bacterial colonization and bacterial eradication, but also for the presence of residual bacteria even within the irrigated area, underscoring the dominant influence of anatomical factors that can limit the effectiveness of bacterial eradication. Both medial and apical regions exhibited higher odds of residual bacterial presence in comparison with the coronal position. Accordingly, the persistence of residual bacteria appears to be primarily position-dependent, highlighting the particular challenge of achieving sufficient disinfection in medial and apical positions, with the anatomical characteristics of the apical dentin possibly contributing to the observed position-dependent persistence of bacteria.

These findings suggest that disinfection measures not only achieve lower areas of bacterial eradication in medial and apical positions, but are also less likely to completely eliminate bacteria in these positions, even within the irrigated area. In this context, reduced areas of bacterial eradication may be interpreted as a limitation in the spatial extent of disinfection (disinfection quantity), whereas the persistence of residual bacteria within the irrigated area may reflect reduced effectiveness or intensity of bacterial elimination (disinfection quality).

## Conclusion

Within the limitations of this study, position within the root canal emerged as the primary determinant of bacterial colonization, bacterial eradication, and the persistence of residual bacteria within the irrigated area, irrespective of the irrigation protocol used — highlighting the predominant influence of anatomical factors on the effectiveness of endodontic disinfection measures. These findings indicate that irrigation protocols should primarily address position-dependent limitations of disinfection measures, particularly in medial and apical positions. Overall, the results of the present study demonstrate that focusing exclusively on the penetration depth of irrigation solutions provides an inadequate understanding of endodontic disinfection — emphasizing the need to adopt new perspectives. Effective disinfection should instead be understood as a combination of the spatial extent (disinfection quantity) and intensity (disinfection quality) of bacterial eradication. Accordingly, future endodontic disinfection strategies should aim to reduce the overall bacterial load by taking into account both the spatial extent and the intensity of bacterial eradication, rather than penetration depth alone.

## Data Availability

The data presented in this study are available on request from the corresponding author.
